# Medical Debt and Deferred Care for Physical Health, Mental Health, and Dental Needs Among U.S. Adults

**DOI:** 10.1007/s11606-026-10215-x

**Published:** 2026-03-10

**Authors:** Kyle J. Moon, Nora V. Becker, Katherine E. M. Miller, Catherine K. Ettman

**Affiliations:** 1 Department of Mental Health, Johns Hopkins Bloomberg School of Public Health, Baltimore, MD, USA, Baltimore, MD, USA; 2 Division of General Medicine, University of Michigan School of Medicine, Ann Arbor, MI, USA; 3 Institute for Health Policy and Innovation, University of Michigan, Ann Arbor, MI, USA; 4 Department of Health Policy and Management, Johns Hopkins Bloomberg School of Public Health, Baltimore, MD, USA; 5 Partnered Evidence-based Policy Resource Center, Boston VA Health Care System, Boston, MA, USA

**Keywords:** Financial hardship, medical, Utilization, health care, Health care seeking behavior, Health insurance, Research, health services

## Abstract

**BACKGROUND:**

Medical debt burdens an estimated 20 million Americans and may contribute to unmet needs for healthcare.

**OBJECTIVE:**

To examine if medical, mental health, or dental needs are differentially sensitive to medical debt and if this varies by type of health insurance.

**DESIGN:**

Cross-sectional, nationally representative survey.

**PARTICIPANTS:**

U.S. adult participants in the 2023 National Health Interview Survey.

**MAIN MEASURES:**

Self-reported medical financial hardship (“medical debt”) and probability of deferred care in the past year for (a) medical, (b) mental health, and (c) dental needs, among adults with medical debt, compared to adults without medical debt.

**KEY RESULTS:**

The overall prevalence of past-year medical debt was 10.7% [95% CI: 10.3, 11.2] and was high across all insurance market segments: 19.5% [17.5, 21.8] among uninsured adults, 12.6% [11.3, 14.1] among adults with Medicaid, 9.3% [8.8, 9.9] among adults with commercial insurance, and 8.1% [7.2, 9.2] among adults with Medicare. Medical debt was associated with a 24.6 [22.4, 26.8] percentage point (pp) increase in the probability of deferred dental care, 17.6 pp [15.9, 19.4] increase in the probability of deferred medical care, and 9.3 pp [7.9, 10.7] increase in the probability of deferred mental healthcare. Associations were largely consistent by health insurance category, although the association between medical debt and deferred medical care was significantly higher (*P* = 0.008) among uninsured adults (32.5 pp [25.6, 39.4]) than adults covered by commercial insurance (16.9 [14.7, 19.1]).

**CONCLUSIONS:**

Medical debt was consistently associated with deferred care, with dental care most commonly deferred, followed by medical care and then mental healthcare. This association remained mostly consistent across all types of health insurance. Policy interventions that aim to address financial barriers to care and the accompanying burden of medical debt may mitigate the health and economic consequences of delayed and forgone care.

## INTRODUCTION

Deferred healthcare is marked by severe consequences, including heightened morbidity and mortality and widening health inequities.^1-6^ The costs of delayed and forgone care are difficult to estimate but often result in greater utilization of high cost resources that could have been averted with routine or preventive services.^2,5-7^ Despite the known and consistent consequences, deferred care is common across the spectrum of health needs, including medical care, mental healthcare, and dental care, due, in large part, to financial barriers to care.^7-11^ U.S. adults face increasing concerns about medical debt when accessing care,^12,13^ which may result in reinforcing adverse outcomes. Indeed, medical debt exacerbates the problem of deferred care,^14-19^ and prior ecological studies highlight the health consequences of medical debt: a higher share of population with any medical debt was associated with poorer physical and mental health as well as higher mortality in a dose–response manner.^20^

While medical debt has been consistently associated with delayed or forgone care,^14-19^ several points of variation have been identified. For one, differences exist in medical debt burden by the type of health condition, with adults with psychiatric conditions being disproportionately affected.^14,21^ Additionally, individuals who are uninsured and living in states that have not expanded Medicaid under the Affordable Care Act bear a disproportionate burden of medical debt.^12,13,22,23^ However, it is important to note that medical debt remains common among commercially insured adults,^12,22,24-26^ especially with high deductible health plans.^16^

Important knowledge gaps in our understanding of medical debt and health service utilization remain. Studies have considered the association of medical debt with deferred care for medical or mental health needs,^14-17^ but studies have not compared which types of care are deferred when someone reports medical debt. Additionally, it is unclear how health insurance coverage may modify the association between medical debt and deferred care. While medical debt may dissuade health services utilization, health insurance coverage may influence the degree of sensitivity to medical debt and care-seeking decisions that may result in medical debt. Past research has demonstrated that adults with fewer assets, including income or savings, are more likely to forgo needed care and may have a higher threshold of illness for engaging the health system.^27-29^ Thus, the association between medical debt and deferred care may be highest among uninsured adults, who likely anticipate higher costs and have a higher threshold for seeking care than adults covered by health insurance. Conversely, if those who are uninsured are already forgoing care,^30^ they may be less sensitive to medical debt than adults who are insured.^14^

In this nationally representative sample of U.S. adults, we estimate the association of medical debt with deferred care for medical, mental health, and dental needs to assess which health needs are more likely to be delayed or forgone due to cost. Additionally, we examine if the association between medical debt and deferred care varies across types of insurance coverage, with the goal of identifying those most affected by medical debt. Findings have implications for future policy interventions to address the growing problem of medical debt and concomitant barriers to care.

## METHODS

### Participants

This cross-sectional study analyzes data from the 2023 National Health Interview Survey (NHIS),^31^ a nationally representative sample of the civilian, noninstitutionalized U.S. adult population. The NHIS sampling strategy has been described elsewhere,^32^ but in brief, the survey includes adults (≥ 18 years of age) with fixed household addresses. People experiencing unsheltered homelessness, people in long-term care facilities, active duty military, and people who are incarcerated are excluded from the sampling frame, but those in emergency shelters, rooming houses, or group homes are included.^33^ In 2023, 53.7% of households completed the initial household roster interview, and of the eligible adults selected for an interview thereafter, 87.6% completed the interview.^33^ The resulting adult sample had a final response rate of 47.0%, comprising 29,522 adults that provided verbal informed consent.^33^ Survey responses were weighted to account for nonresponse and align the study sample with the adult population based on the American Community Survey.

Of the 29,522 adults that participated in 2023, 28,699 (97.2%) had no missing data for the exposure (medical debt) and outcome (deferred care). Less than 3% (*n* = 812) of individuals in our analytic sample had missing data for ≥ 1 covariate, so we used multiple imputation by chained equations to impute missing values, generating 50 imputed datasets.

Secondary analysis of publicly available, deidentified NHIS does not constitute human subjects research, and thus, did not require institutional review board approval. Reporting adheres to the Strengthening the Reporting of Observational Studies in Epidemiology (STROBE) reporting guideline.^34^

### Measures

The primary exposure of interest, medical debt, was ascertained with the following self-reported measure of medical financial hardship, “In the past 12 months, did you have problems paying or were unable to pay any medical bills? Include bills for doctors, dentists, hospitals, therapists, medication, equipment, nursing home or home care,” which is consistent with prior literature on medical debt using national surveys.^14,35,36^ The three outcomes of interest were deferred care for medical, mental health, and dental needs, each of which was a constructed composite variable defined by participants’ self-reports of delaying or forgoing needed care in the past year due to cost, meaning if a participant reported delaying or forgoing care, they were coded as deferring care. Those who reported neither delaying nor forgoing care were coded as not deferring care. Specific questions are reported in Supplemental Table S1.

Sociodemographic characteristics used as covariates included sex (male or female), age (18–24, 25–34, 35–44, 45–54, 55–64, ≥ 65 years), racialized group (non-Hispanic white, non-Hispanic Black, Asian, American Indian or Alaskan Native, Hispanic, other or multiple races), marital status (single, unmarried partnership, married), children in the home (none, ≥ 1 individual younger than 18 years), employment status (employed, unemployed), educational attainment (high school or less, some postsecondary education, bachelor’s degree or higher), health insurance (uninsured, commercial, Medicaid, Medicare advantage, traditional Medicare, dual eligible Medicaid/Medicare, other), household income relative to the federal poverty line [FPL] (below FPL, up to 100–137% FPL, 138–399% FPL, ≥ 400% FPL), medical comorbidities (none, 1 diagnosed health condition, 2 diagnoses, ≥ 3 diagnoses), Census region (Northeast, Midwest, South, West), and the National Center for Health Statistics’ urbanicity designation for the participant’s county of residence (large central metropolitan area, large fringe metropolitan area, medium and small metropolitan area, and nonmetropolitan area).^37^

### Statistical Analysis

We first estimated the prevalence of past-year medical debt, deferred medical care, deferred mental healthcare, and deferred dental care, using sampling weights to generate nationally representative estimates. We then calculated the prevalence of delayed and forgone care, stratified by medical debt exposure. When reporting descriptive statistics, we present unweighted frequencies and weighted percentages.

Next, to evaluate the association of medical debt with delayed and forgone care, we fit a series of survey-weighted logistic regression models. For each outcome, we fit two models: one crude and one adjusted for sociodemographic characteristics. We then estimated average marginal effects and used standard errors that account for sample weighting, stratified cluster sampling, and imputation-associated variance.^38,39^ While marginal effects are used in both observational and experimental studies, our use of the term “effect” does not imply causality and should be interpreted as marginal associations.^40^ Finally, to assess differential associations between medical debt and care-seeking by insurance coverage, we fit models with an interaction term between medical debt and health insurance, using a dichotomous measure of insured versus uninsured. We then fit stratified models to estimate the association of medical debt with deferred care for adults with Medicaid, Medicare (combining traditional and Medicare Advantage), commercial insurance, or no health insurance. We did not fit models for dually eligible patients or “other” insurance due to statistical power constraints and limited interpretability, respectively.

We performed all statistical analyses using R statistical software, version 4.3.1 (R Foundation for Statistical Computing), using the following packages: survey,^41^ mice,^39^ gtsummary,^42^ marginaleffects,^43^ and EValue.^44^ Statistical tests were 2-sided, with significance defined as *P* < 0.05.

### Sensitivity analysis

We performed several robustness checks. First, recognizing differences in the distribution of characteristics for adults with and without medical debt, we calculate marginal effects at the mean values. Second, we assessed the imputation procedure by using a complete case analysis, restricting the sample to the 27,887 individuals with no missing data, and compared estimates to those obtained with multiple imputation. Third, we considered differences between delaying and forgoing care, using each as a separate outcome rather than a composite outcome. Fourth, to account for unobserved confounding, we calculated e-values to quantify how much confounding would be required to negate the observed associations.^45,46^ Finally, we fit models that adjust for dental insurance coverage, in addition to health insurance.

## RESULTS

Among 28,699 adults surveyed in 2023, 2,835 (10.7% [95% CI: 10.3, 11.2]) reported past-year medical debt. The prevalence of medical debt was highest among those who were uninsured (*n* = 382; 19.5% [17.5, 21.8]), followed by adults covered by Medicaid (*n* = 368; 12.6% [11.3, 14.1]), commercial insurance (*n* = 1,426; 9.3% [8.8, 9.9]), and Medicare (*n* = 366; 8.1 [7.2, 9.2]). The majority of participants were non-Hispanic white (62%), ≥ 45 years of age (54%), female (51%), married (51%), residents of large metropolitan counties (55%), employed (64%), covered by commercial health insurance plans (61%), and had been diagnosed with ≥ 1 chronic medical conditions (64%). Sociodemographic characteristics are presented in Table 1.

**Figure 1:**
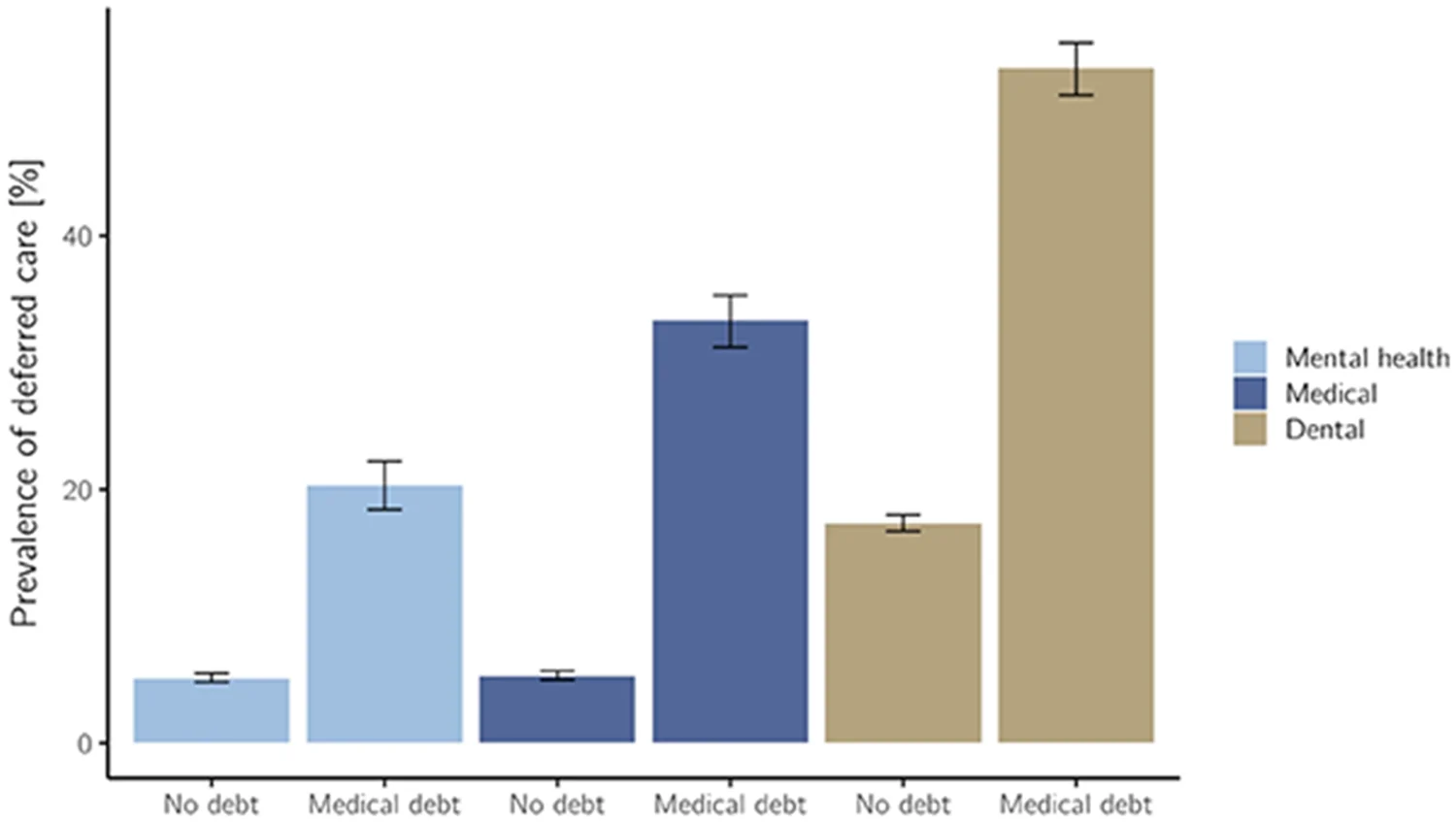
Prevalence of past-year deferred care, by past-year medical debt exposure. Deferred mental healthcare is shown in light blue, deferred medical care is shown in blue, and deferred dental care is shown in gold. Estimates are unadjusted survey-weighted prevalence estimates with 95% confidence intervals. Data source: National Center for Health Statistics, National Health Interview Survey, 2023.

In unadjusted analyses, roughly one-third of U.S. adults with medical debt reported deferring medical care in the past year due to cost (33.3% [95% CI: 31.2, 35.3]), compared to 5.3% [5.0, 5.7] of adults without medical debt, corresponding to more than a sixfold increase in the prevalence of deferred medical care. Similarly, the unadjusted prevalence of deferred mental healthcare was fourfold higher among adults with medical debt (20.3% [18.4, 22.2]), compared to adults without medical debt (5.1 [4.8, 5.5]). More than half of adults with medical debt (53.2% [51.1, 55.2]) reported deferring dental care, which is more than three times as high than the unadjusted prevalence of deferred dental care among those without medical debt (17.3 [16.7, 18.0]), shown in Fig. 1.

**Figure 2:**
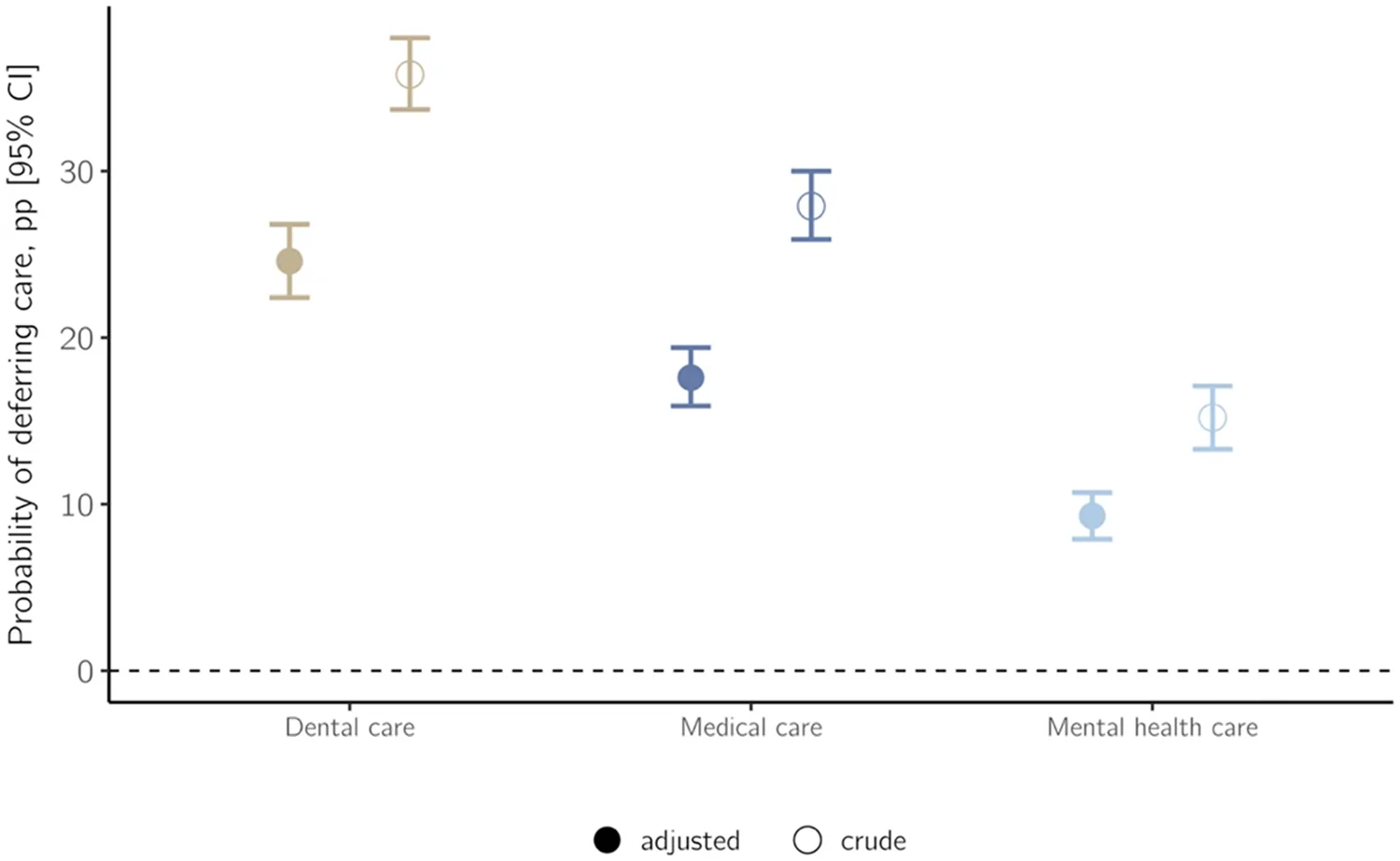
Association of medical debt with probability of deferred care, relative to adults with no medical debt. Estimates are presented as average marginal effects with 95% confidence intervals (pp = percentage point). Closed circles are estimates obtained from models that adjust for sociodemographic characteristics, and open circles are estimates obtained from crude (unadjusted) models. The dotted vertical line represents the reference group (adults with no medical debt). Data source: National Center for Health Statistics, National Health Interview Survey, 2023.

After adjusting for sociodemographic characteristics, past-year medical debt was associated with a 24.6 percentage point (pp) increase in the probability of deferred dental care [95% CI: 22.4, 26.8], a 17.6 pp [15.9, 19.4] increase in the probability of deferred medical care, and a 9.3 pp [7.9, 10.7] increase in the probability of deferred mental healthcare (Fig. 2). Point estimates for crude and adjusted models are presented in Table S2.

**Figure 3:**
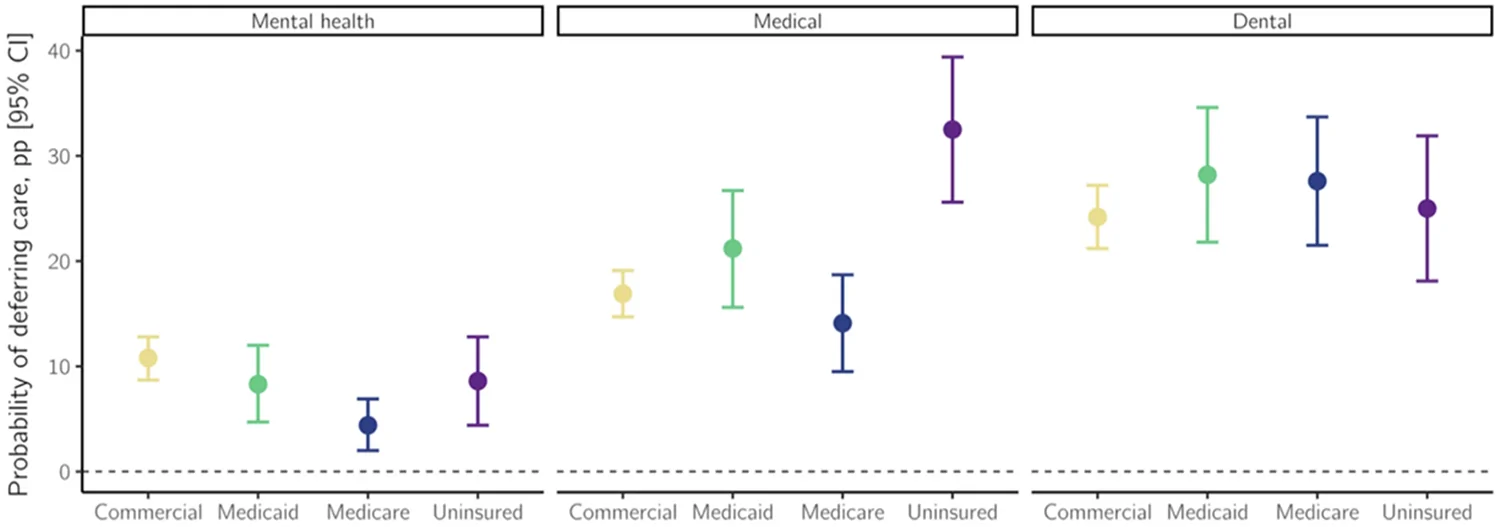
Association of medical debt with probability of deferred care, relative to adults with no medical debt, stratified by health insurance coverage. The three panel figure shows the association between medical debt and the probability of deferred care for (a) mental health needs (left), (b) medical needs (center), and (c) dental needs (right). All estimates are presented as average marginal effects, using multivariable models that adjust for sociodemographic characteristics of the respondents. Purple points represent those that reported being uninsured, blue points represent those covered by Medicare, green points represent those covered by Medicaid, and yellow points represent those covered by commercial insurance. pp = percentage point. Data source: National Center for Health Statistics, National Health Interview Survey, 2023.

Associations between medical debt and deferred care were largely consistent across insurance coverage, with the greatest magnitude of association for deferred dental care, followed by deferred medical care, and then deferred mental healthcare across all types of insurance coverage (Fig. 3). In tests of effect measure modification, we found a significant difference in the magnitude of the association between medical debt and deferred medical care for those who are insured vs. uninsured (*P* = 0.008); medical debt was associated with a higher probability of deferred care among uninsured adults (32.5 pp [25.6, 39.4]) than commercially insured adults (16.9 [14.7, 19.1]) but not among those covered by Medicaid or Medicare. Tests of effect measure modification were not statistically significant for mental healthcare (*P* = 0.22) or dental care (*P* = 0.05), with comparable associations between medical debt and deferred care across all four insurance groups (uninsured, Medicare, Medicaid, and commercial), demonstrated by overlapping confidence intervals. Point estimates for crude and adjusted models, stratified by insurance coverage, are presented in Table S3.

### Sensitivity analyses

Findings were robust to several analyses assessing sensitivity to the imputation procedure (Table S4), differences in the distribution of characteristics between adults with and without medical debt (Table S5), differences between delayed and forgone care (Table S6), omitted variable bias (Supplemental Results), and adjustment for dental insurance (Table S7).

## DISCUSSION

In this nationally representative sample of U.S. adults, about 1 in 7 individuals reported medical financial hardship in the past year. The prevalence of self-reported inability to pay medical bills, a common measure for medical debt, was markedly higher among those who were uninsured (1 in 5 adults), followed by those covered by Medicaid (1 in 8 adults), commercial insurance (1 in 11 adults), and Medicare (1 in 12 adults), although such estimates are confounded by income. Deferred care was common among all adults, especially for dental care (1 in 5 adults), followed by medical care (1 in 11 adults), and mental healthcare (1 in 15 adults). Across all types of insurance coverage, medical debt was consistently associated with deferred care for medical, mental health, and dental needs, but to varying extents. Dental care was most often deferred among those with medical debt, followed by medical care and then mental healthcare. Compared to those covered by commercial insurance, uninsured adults were more likely to defer medical care when faced with medical debt. However, the probability of deferred care for both mental health and dental needs were comparable among those who are insured and uninsured. Taken together, these results suggest that medical debt contributes to unmet need for treatment across the spectrum of health needs for all American adults, irrespective of insurance coverage, and dental care might be the most sensitive to medical debt.

In terms of the prevalence of medical debt, our estimate of 11% is consistent with prior literature ranging from 11 to 18%.^12,13,15,47^ Prior research has noted that the following groups bear greater burdens of medical debt: women, non-Hispanic Black adults, low- and middle-income individuals, individuals with medical and psychiatric conditions, and uninsured individuals,^12,14,21,23,47,48^ all of which are consistent with our findings. The heightened prevalence of medical debt among the uninsured highlights the important role of health insurance in ensuring financial security and the need for continued efforts to reduce the number of uninsured Americans.^26,49^ However, medical debt is still common among the insured population,^22^ which raises important questions about the quality of current health plans. Indeed, consistent associations between medical debt and deferred care across all types of insurance coverage underscore the need to address the affordability of care, even among the insured,^21,50^ and promote access to assets.^51^

This study is subject to several limitations. First, wide variability exists in how past studies have defined medical debt, influencing prevalence estimates.^52^We use a self-reported measure of medical financial hardship, which may not fully capture medical debt.^52^ Additionally, both the exposure and outcome were self-reported and may suffer from recall bias. However, perceived debt may have a greater influence on care-seeking decisions than actual debt.^15^ Second, this study is cross-sectional, hindering our ability to account for the temporality of exposure and outcome, ensuring that medical debt preceded care-seeking decisions. Thus, we cannot establish causality. Third, we cannot differentiate between newly acquired and existing medical debt, and considering medical debt can follow people for years,^53^ survey responses may not represent debt incurred with their current health insurance coverage. Fourth, all respondents were asked about deferred care, but there were no questions about perceived need for medical, mental health, or dental care. Fifth, medical debt was a dichotomous measure of medical financial hardship, hindering our ability to assess differences in amounts of debt. While we observed a higher prevalence of medical debt among Medicaid beneficiaries compared to those covered by commercial insurance, the amount of medical debt likely differs, as cost-sharing is low in the Medicaid program.^54^ Finally, due to limited statistical power, we did not fit stratified models for dually eligible patients, a population that is medically complex and may experience outsized impacts of medical debt. Further studies are needed to explore the prevalence and consequences of medical debt in this population as well as consider differences by Medicare plan (traditional vs. Medicare Advantage).

Notwithstanding these limitations, this study adds to the growing literature on medical debt and its consequences for health services utilization. When comparing which types of care tend to get deferred among those with a history of medical debt, we found that dental care is the most commonly deferred, followed by medical care and then mental healthcare. This finding aligns with prior studies reporting that more adults report financial barriers to accessing dental care than any other type of healthcare.^10^ Past work has found that, regardless of age, income, or type of insurance, U.S. adults face greater financial barriers to dental care than any other health care,^10^ consistent with our findings. Dental care is often a separate benefit for commercially-insured patients, excluded from traditional Medicare, and widely variable across Medicare Advantage plans and state Medicaid programs. However, even when accounting for coverage by a separate dental plan, dental needs remain the most sensitive to medical debt, warranting further research to better understand why dental needs may be prioritized differently from medical or mental health care. This sensitivity of dental care to medical debt is consistent with evidence from the Oregon Health Insurance Experiment, which found that Medicaid expansion had significant effects on dental care utilization, reducing unmet need for dental care.^55^

There was a robust association between medical debt and deferred mental healthcare across insurance market segments, which is consistent with prior studies.^14,15^ However, it remains unclear why mental healthcare would be the least sensitive to medical debt, as past studies have reported that the demand for mental healthcare is more elastic (i.e., sensitive to prices) than medical care.^56-58^ Compared to dental and medical care, a smaller subgroup of people likely consume mental healthcare at baseline. This could be explained by a greater number of people, irrespective of medical debt exposure, deferring mental healthcare due to stigma, limited coverage, poor network adequacy, or long wait-times,^59-63^ but further inquiry is needed. When assessing how the association between medical debt and deferred care varies by insurance status, we found that estimates were largely consistent across insured and uninsured adults. This underscores the far-reaching impacts of medical debt, exacerbating treatment delays that may cascade into other health issues, ^2-4,64,65^ result in greater utilization of high cost resources,^2,5,6^ and produce high individual and societal costs.

Addressing the growing toll of medical debt may provide an important strategy in mitigating the health and economic consequences of deferred care. In this nationally representative sample of U.S. adults, medical debt was consistently associated with deferred care for medical, mental health, and dental needs across insurance market segments. Recent policy developments, such as historic cuts to limit insurance coverage in the 2025 Budget Reconciliation Act, may exacerbate the problem of medical debt and its collateral health and economic consequences of deferred care. Policies that address the affordability of care and concomitant challenges with medical debt may aid in improving health.

## Supplementary Material

Supplemental Material

**Supplementary Information** The online version contains supplementary material available at https://doi.org/10.1007/s11606-026-10215-x.

## Figures and Tables

**Table 1:** Sociodemographic Characteristics of NHIS 2023 Respondents, By Medical Debt Exposure^a,b^

	Overall (*n* = 28,699)	No Medical Debt (*n* = 25,864)	Medical Debt (*n* = 2,835)
Sex			
Male	13,102 [49%]	11,968 [49%]	1,134 [44%]
Female	15,592 [51%]	13,893 [51%]	1,699 [56%]
Age			
18–24 years	1,810 [12%]	1,637 [12%]	173 [11%]
25–34 years	4,140 [17%]	3,678 [17%]	462 [20%]
35–44 years	4,411 [17%]	3,891 [17%]	520 [19%]
45–54 years	3,950 [15%]	3,440 [15%]	510 [19%]
55–64 years	4,903 [16%]	4,347 [16%]	556 [17%]
≥ 65 years	9,427 [23%]	8,819 [24%]	608 [14%]
Race and ethnicity			
Non-Hispanic white	19,058 [62%]	17,367 [63%]	1,691 [57%]
Asian	1,600 [6%]	1,528 [7%]	72 [3%]
American Indian or Alaska Native	190 [1%]	172 [1%]	18 [1%]
Black	3,057 [12%]	2,594 [11%]	463 [17%]
Hispanic	4,274 [17%]	3,751 [17%]	523 [21%]
Other ^c^	520 [2%]	452 [2%]	68 [2%]
Education			
High school or less	9,697 [37%]	8,547 [37%]	1,150 [45%]
Some college	7,941 [29%]	6,965 [29%]	976 [35%]
Bachelor’s degree or higher	10,931 [33%]	10,239 [35%]	692 [20%]
Employment Status			
Unemployed	11,841 [36%]	10,703 [36%]	1,138 [37%]
Employed	16,311 [64%]	14,665 [64%]	1,646 [63%]
Annual household income relative to the federal poverty line (FPL)			
Below FPL	2,983 [10%]	2,524 [9%]	459 [15%]
100–137% FPL	1,947 [7%]	1,596 [6%]	351 [12%]
138–399% FPL	11,861 [41%]	10,424 [40%]	1,437 [51%]
≥ 400% FPL	11,908 [42%]	11,320 [44%]	588 [23%]
Marital status			
Married	12,911 [51%]	11,849 [52%]	1,062 [45%]
Unmarried partnership	1,877 [9%]	1,650 [9%]	227 [11%]
Single	13,382 [40%]	11,887 [39%]	1,495 [44%]
Children (aged < 18 years) in the home			
No	21,448 [68%]	19,527 [69%]	1,921 [61%]
Yes	7,251 [32%]	6,337 [31%]	914 [39%]
Insurance ^d^			
Uninsured	1,964 [8%]	1,582 [8%]	382 [15%]
Commercial	16,716 [61%]	15,290 [62%]	1,426 [53%]
Medicaid	2,891 [12%]	2,523 [12%]	368 [15%]
Medicare Advantage	3,492 [8%]	3,224 [9%]	268 [6%]
Traditional Medicare	1,040 [2%]	942 [3%]	98 [2%]
Dual eligible	751 [2%]	669 [2%]	82 [2%]
Other	1,707 [5%]	1,509 [5%]	198 [6%]
Urbanicity			
Large central metro	8,584 [30%]	7,830 [31%]	754 [27%]
Large metro fringe	6,654 [25%]	6,010 [25%]	644 [26%]
Small metro	9,023 [30%]	8,043 [30%]	980 [33%]
Nonmetro	4,438 [14%]	3,981 [14%]	457 [14%]
U.S. Census region			
Northeast	4,452 [17%]	4,090 [18%]	362 [14%]
Midwest	6,315 [21%]	5,690 [21%]	625 [20%]
South	10,663 [38%]	9,389 [37%]	1,274 [47%]
West	7,269 [24%]	6,695 [24%]	574 [19%]
Comorbidities			
0 diagnosed conditions	8,851 [36%]	8,285 [37%]	566 [24%]
1 diagnosed condition	6,803 [24%]	6,295 [25%]	508 [19%]
2 diagnosed conditions	5,427 [18%]	4,905 [17%]	522 [19%]
≥ 3 diagnosed conditions	7,618 [23%]	6,379 [21%]	1,239 [38%]

a Data source: National Center for Health Statistics, National Health Interview Survey, 2023

b Results are presented as unweighted frequencies [weighted percentages]

c Other race/ethnicity includes individuals coded as multiple races and other single

d In stratified models, we combined Medicare Advantage and Traditional Medicare into one category

## Data Availability

Data are publicly available from the U.S. Centers for Disease Control and Prevention: https://www.cdc.gov/nchs/nhis/documentation/2023-nhis.html.
