# Supplementary material for: Medical Debt and Deferred Care for Physical Health, Mental Health, and Dental Needs Among U.S. Adults
Source: J Gen Intern Med. Author manuscript; Available in PMC 2026 Mar 28. (PMC13032188; doi:10.1007/s11606-026-10215-x)
Supplement: Supplemental Material [file NIHMS2155575-supplement-Supplemental_Material.docx]

**SUPPLEMENTARY MATERIALS**

**Supplemental Results**

We repeated the main analysis with a complete case analysis (n = 27,887) to assess the multiple imputation procedure, which yielded comparable estimates (**Table S4**). To assume the same distribution of characteristics for adults with and without medical debt, we calculated marginal effects at the mean values, which can be interpreted as the association of medical debt with the probability of deferring care for the adult with the average characteristics in the sample (**Table S5**). This approach yielded qualitatively the same findings; the association between medical debt and deferred care was greatest in magnitude for dental care, followed by medical care, and then mental healthcare. Additionally, we found no substantive differences between delaying and forgoing care within a given type of care. For example, estimates between delayed mental healthcare and forgone mental healthcare were comparable, as were estimates for delayed and forgone medical care as well as delayed and forgone dental care (**Table S6**).

To assess sensitivity to omitted variable bias, we estimated e-values for each outcome. For deferred medical care, we estimated an e-value of 12.1, meaning an unmeasured confounder would need to be associated with both medical debt and deferred medical care by 12.1-fold, conditional on the observed covariates, to fully explain away the observed association between medical debt and deferred medical care. ^45^ Similarly, the observed association of medical debt with deferred mental health and dental care could be explained away by an unmeasured confounder associated with both the exposure and outcome by 6.8-fold and 3.4-fold each, respectively, but weaker confounding could not do so.^45^ To put these e-values into context,^46^ the strongest predictors for deferred medical, mental health, and dental care had odds ratios of 6.3, 4.7, and 3.9, respectively. While e-values differed in magnitude across outcomes, all estimated e-values are larger than the strongest predictors of each outcome, which signifies robustness to potential unmeasured confounding.

**Table S1. Survey measures of delayed and forgone care.**

| **Composite** | **Item** | **Question** |
| --- | --- | --- |
| Deferred medical care | Delayed medical care | During the past 12 months, have you DELAYED getting medical care because of the cost? |
|  | Forgone medical care | During the past 12 months, was there any time when you needed medical care, but DID NOT GET IT because of the cost? |
| Deferred mental healthcare | Delayed mental healthcare | During the past 12 months, have you DELAYED getting counseling or therapy from a mental health professional because of the cost? |
|  | Forgone mental healthcare | During the past 12 months, was there any time when you needed counseling or therapy from a mental health professional, but DID NOT GET IT because of the cost? |
| Deferred dental care | Delayed dental care | During the past 12 months, have you DELAYED getting dental care because of the cost? |
|  | Forgone dental care | During the past 12 months, was there any time when you needed dental care, but DID NOT GET IT because of the cost? |

**Table S2. Association of medical debt with deferred care for medical, mental health, and dental needs.** ^a,b^

| **Outcome** | **Medical debt** | **n** | **Weighted % [95% CI]** ^c^ | **Unadjusted AME** ^d^ **[95% CI]** | **Adjusted AME** ^d^ **[95% CI]** |
| --- | --- | --- | --- | --- | --- |
| Deferred medical care | No | 1,258 | 5.3  [5.0, 5.7] | 0  [reference] | 0  [reference] |
|  | Yes | 946 | 33.3  [31.2, 35.3] | 27.9  [25.9, 30.0] | 17.6  [15.9, 19.4] |
| Deferred mental healthcare | No | 1,264 | 5.1  [4.8, 5.5] | 0  [reference] | 0  [reference] |
|  | Yes | 563 | 20.3  [18.4, 22.2] | 15.2  [13.3, 17.1] | 9.3  [7.9, 10.7] |
| Deferred dental care | No | 4,309 | 17.3  [16.7, 18.0] | 0  [reference] | 0  [reference] |
|  | Yes | 1,516 | 53.2  [51.1, 55.2] | 35.8  [33.7, 38.0] | 24.6  [22.4, 26.8] |

^a^ Data source: National Center for Health Statistics, National Health Interview Survey, 2023.

^b^ These point estimates are shown graphically in Figure 2 of the main text.

^c^ Weighted % refers to the weighted prevalence of deferred care.

^d^ AME = average marginal effect

**Table S3. Association of medical debt with deferred care for medical, mental health, and dental needs, stratified by health insurance coverage of respondents.** ^a,b^

|  | Uninsured (n = 1,964) | | Commercial (n = 16,716) | | Medicaid (n = 2,891) | | Medicare (n = 4,532) | |
| --- | --- | --- | --- | --- | --- | --- | --- | --- |
|  | Crude AME [95% CI] ^c^ | Adjusted AME [95% CI] ^d^ | Crude AME [95% CI] | Adjusted AME [95% CI] | Crude AME [95% CI] | Adjusted AME [95% CI] | Crude AME [95% CI] | Adjusted AME [95% CI] ^e^ |
| Medical | 35.5  [28.8, 42.2] | 32.5  [25.6, 39.4] | 26.2  [23.6, 28.9] | 16.9  [14.7, 19.1] | 22.9  [17.5, 28.4] | 21.2  [15.6, 26.7] | 19.1  [14.2, 24] | 14.1  [9.5, 18.7] |
| Mental health | 16.0  [11.0, 20.9] | 8.6  [4.4, 12.8] | 16.9  [14.3, 19.5] | 10.8  [8.7, 12.8] | 11.9  [7.3, 16.5] | 8.3  [4.7, 12.0] | 7.0  [3.7, 10.4] | 4.4  [2.0, 6.9] |
| Dental | 29.8  [23.5, 36.1] | 25.0  [18.1, 31.9] | 34.3  [31.4, 37.3] | 24.2  [21.2, 27.2] | 31.6  [25.4, 37.9] | 28.2  [21.8, 34.6] | 34.1  [27.8, 40.4] | 27.6  [21.5, 33.7] |

^a^ Data source: National Center for Health Statistics, National Health Interview Survey, 2023.

^b^ These point estimates are presented graphically in Figure 3 of the main text.

^c^ AME = average marginal effect

^d^ Adjusted for participant sex, race, age, income relative to federal poverty line, employment status, educational attainment, marital status, whether any children (aged < 18 years) live in the home, number of medical comorbidities, urbanicity designation of the county of residence, and Census region.

^e^ For Medicare models, we used all of the same covariates as above, except age category because only adults ≥ 65 years of age were included in this sample, as dual eligible adults were excluded from this category.

**Table S4. Association of medical debt with deferred care, using complete case analysis (n = 27,887).**

| **Outcome** | **AME ^b^** | **Lower 95% CI ^c^** | **Upper 95% CI ^c^** |
| --- | --- | --- | --- |
| Deferred medical care | 18.6 | 16.8 | 20.4 |
| Deferred mental healthcare | 9.8 | 8.3 | 11.3 |
| Deferred dental care | 24.7 | 22.6 | 26.9 |

^a^ AME = average marginal effect

^b^ CI = confidence interval

**Table S5. Association of medical debt with deferred care, adjusted for sociodemographic characteristics, using marginal effects at mean values.**

|  | MEM ^a^ | Lower 95% CI ^b^ | Upper 95% CI ^b^ |
| --- | --- | --- | --- |
| Deferred medical care | 7.2 | 5.4 | 9.0 |
| Deferred mental healthcare | 2.5 | 1.8 | 3.3 |
| Deferred dental care | 13.0 | 11.0 | 14.9 |

^a^ MEM = marginal effect at the mean

^b^ CI = confidence interval

**Table S6. Association of medical debt with delayed and forgone care.**

| **Outcome** | **Medical debt** | **Weighted % [95% CI]** | **Crude AME [95% CI]** ^a^ | **Adjusted AME [95% CI]** ^a^ |
| --- | --- | --- | --- | --- |
| Delayed medical care | No | 4.4%  [4.1, 4.8] | 0  [reference] | 0  [reference] |
|  | Yes | 29.6%  [27.7, 31.6] | 25.2  [23.2, 27.2] | 15.8  [14.2, 17.4] |
| Forgone medical care | No | 3.8%  [3.5, 4.2] | 0  [reference] | 0  [reference] |
|  | Yes | 27.0%  [25.1, 28.9] | 23.1  [21.2, 25.1] | 13.8  [12.3, 15.3] |
| Delayed mental healthcare | No | 4.4%  [4.1, 4.7] | 0  [reference] | 0  [reference] |
|  | Yes | 17.8%  [16.1, 19.8] | 13.4  [11.6, 15.3] | 8.3  [7.0, 9.6] |
| Forgone mental healthcare | No | 4.0%  [3.7, 4.3] | 0  [reference] | 0  [reference] |
|  | Yes | 18.1%  [16.4, 20.0] | 14.2  [12.4, 16.0] | 8.3  [7.0, 9.6] |
| Delayed dental care | No | 15.3%  [14.7, 15.9] | 0  [reference] | 0  [reference] |
|  | Yes | 49.1%  [47.0, 51.2] | 33.8  [31.7, 36.0] | 22.8  [20.7, 24.9] |
| Forgone dental care | No | 12.0%  [11.5, 12.6] | 0  [reference] | 0  [reference] |
|  | Yes | 42.1%  [40.0, 44.3] | 30.1  [27.9, 32.3] | 18.9  [16.9, 20.9] |

^a^ AME = average marginal effect

**Table S7. Association of medical debt with deferred care for dental needs, adjusted for dental insurance status.**

|  | AME ^a^ | Lower 95% CI ^b^ | Upper 95% CI ^b^ |
| --- | --- | --- | --- |
| Model 1: includes a binary indicator of separate dental plan ^c,d^ | 24.7 | 22.6 | 26.9 |
| Model 2: includes an interaction term between medical debt and separate dental insurance plan ^c,e^ | 24.7 | 22.5 | 26.9 |

^a^ AME = average marginal effect

^b^ CI = confidence interval

^c^ Models also adjusted for the same covariates as models reported in the main text: sex, age, race and ethnicity, annual household income relative to the federal poverty line, educational attainment, employment status, children in household, health insurance status (binary indicator of insured vs. uninsured), marital status, medical comorbidities, Census region, and urbanicity.

^d^ The binary indicator was constructed using responses to the following question: “Are you covered by a SEPARATE plan that only pays for dental services?”

^e^ We found no evidence of differences in the magnitude of association between medical debt and deferred dental care for those who have a separate dental insurance plan vs. those who do not have a separate dental insurance plan (*P* = 0.67).
